# Photodynamic Therapy for the Treatment and Diagnosis of *Cancer*–A Review of the Current Clinical Status

**DOI:** 10.3389/fchem.2021.686303

**Published:** 2021-08-02

**Authors:** Gurcan Gunaydin, M. Emre Gedik, Seylan Ayan

**Affiliations:** ^1^ Department of Basic Oncology, Hacettepe University Cancer Institute, Ankara, Turkey; ^2^ Department of Chemistry, Bilkent University, Ankara, Turkey

**Keywords:** PDT–photodynamic therapy, tumor, clinical application, PDD–photodynamic diagnosis, combination (combined) therapy, photoimmunotherapy (PIT)

## Abstract

Photodynamic therapy (PDT) has been used as an anti-tumor treatment method for a long time and photosensitizers (PS) can be used in various types of tumors. Originally, light is an effective tool that has been used in the treatment of diseases for ages. The effects of combination of specific dyes with light illumination was demonstrated at the beginning of 20th century and novel PDT approaches have been developed ever since. Main strategies of current studies are to reduce off-target effects and improve pharmacokinetic properties. Given the high interest and vast literature about the topic, approval of PDT as the first drug/device combination by the FDA should come as no surprise. PDT consists of two stages of treatment, combining light energy with a PS in order to destruct tumor cells after activation by light. In general, PDT has fewer side effects and toxicity than chemotherapy and/or radiotherapy. In addition to the purpose of treatment, several types of PSs can be used for diagnostic purposes for tumors. Such approaches are called photodynamic diagnosis (PDD). In this Review, we provide a general overview of the clinical applications of PDT in cancer, including the diagnostic and therapeutic approaches. Assessment of PDT therapeutic efficacy in the clinic will be discussed, since identifying predictors to determine the response to treatment is crucial. In addition, examples of PDT in various types of tumors will be discussed. Furthermore, combination of PDT with other therapy modalities such as chemotherapy, radiotherapy, surgery and immunotherapy will be emphasized, since such approaches seem to be promising in terms of enhancing effectiveness against tumor. The combination of PDT with other treatments may yield better results than by single treatments. Moreover, the utilization of lower doses in a combination therapy setting may cause less side effects and better results than single therapy. A better understanding of the effectiveness of PDT in a combination setting in the clinic as well as the optimization of such complex multimodal treatments may expand the clinical applications of PDT.

## Introduction

**FIGURE 1:**
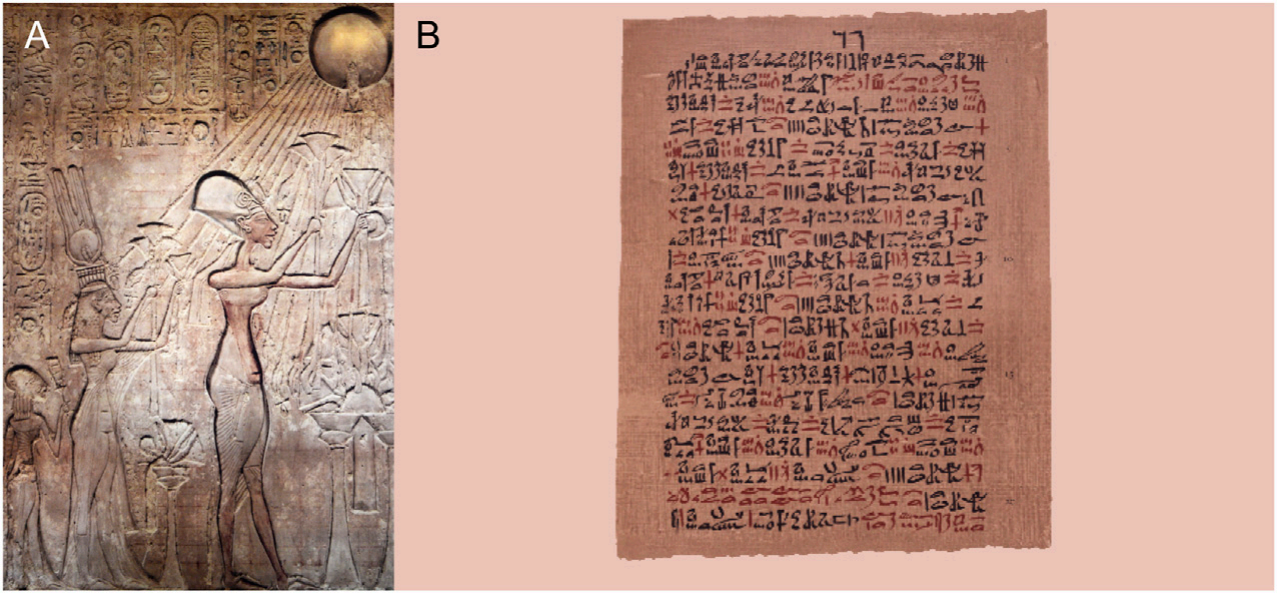
Phototherapy in Ancient Egypt. **(A)** Veneration of the sun eventually resulted in its utilization as heliotherapy (Shutterstock). **(B)** The Ebers Papyrus mentioned the Pharaoh’s utilization of phototherapy utilizing various plants (Ebers papyrus, University of Leipzig, Germany, https://commons.wikimedia.org, public domain) (Abdel-kader, 2016).

Light is an effective tool that has been used in the treatment of maladies for ages. It was utilized in the treatment of skin diseases in ancient Egypt, India, and China. In addition, photochemotherapy has been used as a treatment method for a long time (Roelandts, 1991). It is known that psoralens were used in India around 1400 BC. In *Atharva Veda*, which is a collection of Vedic Sanskrit incantations, spells, and hymns, it is stated that Hindus used the psoralens obtained from the seeds of *Psoralea corylifolia* for the repigmentation of the vithlogenic skin, utilizing the ancient Ayurvedic medicine (Fitzpatrick and Pathak, 1959; Merriam-Webster, 2021). In Ancient Egypt, the Sun represented an important part of the culture and sunlight could be associated with healing properties (Honigsmann, 2013; Grzybowski et al., 2016). The importance of the Sun in Ancient Egypt resulted in its utilization in terms of heliotherapy (Figure 1A) (Goldberg, 1930; McDonagh, 2001). Figure 1 demonstrates phototherapy in Ancient Egypt. The Ebers papyrus (a total length of 20 m) represents the largest record of Ancient Egyptian medicine (Hartmann, 2016) and the Papyrus (circa 1500 BC) mentions about vitiligo (Figure 1B) (Ebers, 1875; Bryan, 1930; Millington and Levell, 2007). It is known that Egyptians extracted psoralen from the *Ammi majus* (bishop’s weed) plant growing on the south bank of the Nile and used it in the treatment of leukoderma in the 12th century AD (Daniell and Hill, 1991). Heliotherapy (the use of sunlight or of another source of UV, visible or infrared radiation for therapeutic purposes), which was systematically recorded and described to be used for medicinal purposes by Herodotus in ancient Greece, was applied as an effective treatment method for various diseases (Mitton and Ackroyd, 2008; Honigsmann, 2013; Ceglia and Toni, 2018). In the 18th and 19th centuries, sunlight therapy was used to treat various ailments such as *tuberculosis*, rickets, scurvy, rheumatism, paralysis, edema, and muscle weakness. However, its utilization in modern medicine started in the 20th century. At the beginning of the 20th century, Danish physician Niels Ryberg Finsen reported that he treated chickenpox under red light by preventing inflammation of the pustules. In the following years, he was awarded with the 1903 Nobel Prize in Physiology or Medicine for “his contribution to the treatment of diseases, especially lupus vulgaris, with concentrated light radiation” (NobelPrize.org; Urbach et al., 1976; Ackroyd et al., 2001).

## History of Photodynamic Therapy

The idea of using photodynamic therapy (PDT) as a new treatment strategy was suggested in the early 1900s. In fact, the extraction of hematoporphyrin from dried blood by Schere via removing iron in 1841 is an important milestone for the field of photosensitizers (PSs) (Rasmussen-Taxdal et al., 1955; Kou et al., 2017). A researcher named Oscar Raab observed that paramecia died when incubated under the light with acridine red dyes in Germany. He also observed that those that were not exposed to light remained alive (Hamblin, 2018). In fact, initial observations related with PDT in medicine refer to the inactivation of microorganisms more than a hundred years ago (Raab, 1900; Jori et al., 2006). Thus, the definition of photodynamic action emerged based on this observation. Indeed, Hermann von Tappeiner coined the term “photodynamic reaction”, underlining the fact that light played an important role (v. Tappeiner, 1909; Niculescu and Grumezescu, 2021). PDT of infections, which involves reactive oxygen species (ROS) to disrupt and kill microbes, can be useful against bacteria and viruses (Hamblin and Hasan, 2004; Jori, 2006; Oyim et al., 2021; Rapacka-Zdonczyk et al., 2021; Warrier et al., 2021). Due to the World Wars, the evolution of the idea has been delayed for nearly 60 years.

In 1960, generation of new approaches in terms of PDT started with the studies by R. L. Lipson and S. Schwartz at the Mayo Clinic (Dougherty et al., 1998). Lipson *et al.* reported that malignant diseases could be detected by using an acetic acid-sulfuric acid derivative of hematoporphyrin and proper filter systems for activating and viewing the fluorescence (Lipson and Baldes, 1960; Lipson et al., 1961). Schwartz treated hematoporphyrin with acetic acid and sulfuric acid and obtained a porphyrin mixture in order to achieve optimal tumor localization. He termed this mixture as hematoporphyrin derivative (HpD), which contains several porphyrins, monomers as well as dimers and oligomers (Moan, 1986). Lipson *et al.* used HpD in order to detect tumors (Dougherty et al., 1998). In 1967, Lipson *et al.* reported the use of hematoporphyrin derivative in patients for lesions of the esophagus and tracheobronchial tree, cervix and vagina, rectum, breast, tonsil, parotid and intraperitoneal lesions. They proposed that this approach could be helpful in terms of detection and subsequent management of malignant diseases (Lipson et al., 1967). The utilization of HpD in bladder cancer treatment was reported by Kelly and Snell as one of the first clinical applications in 1976 (Dougherty, 1996; Dougherty et al., 1998; Abrahamse and Hamblin, 2016). In the following years, HpD was transformed into a drug formulation by Dougherty. Dougherty *et al.* reported the first clinical case of PDT in a patient with metastatic breast cancer to the skin in 1978 (Dougherty et al., 1998). Furuse *et al.* conducted phase II clinical trials using Photofrin (porfimer sodium) for early-stage lung cancer between June 1989 and February 1992 (Furuse et al., 1993). In 1993, PDT with Photofrin obtained the first health agency approval in Canada for the treatment of bladder cancer (Usuda et al., 2006). The Food and Drug Administration (FDA) approved Photofrin for esophageal cancer in 1995. In addition, Photofrin was approved for the treatment of early non-small cell lung cancer in 1998 (Pass, 1993; Dougherty et al., 1998; Kato, 2004; Huang, 2005).

**FIGURE 2:**
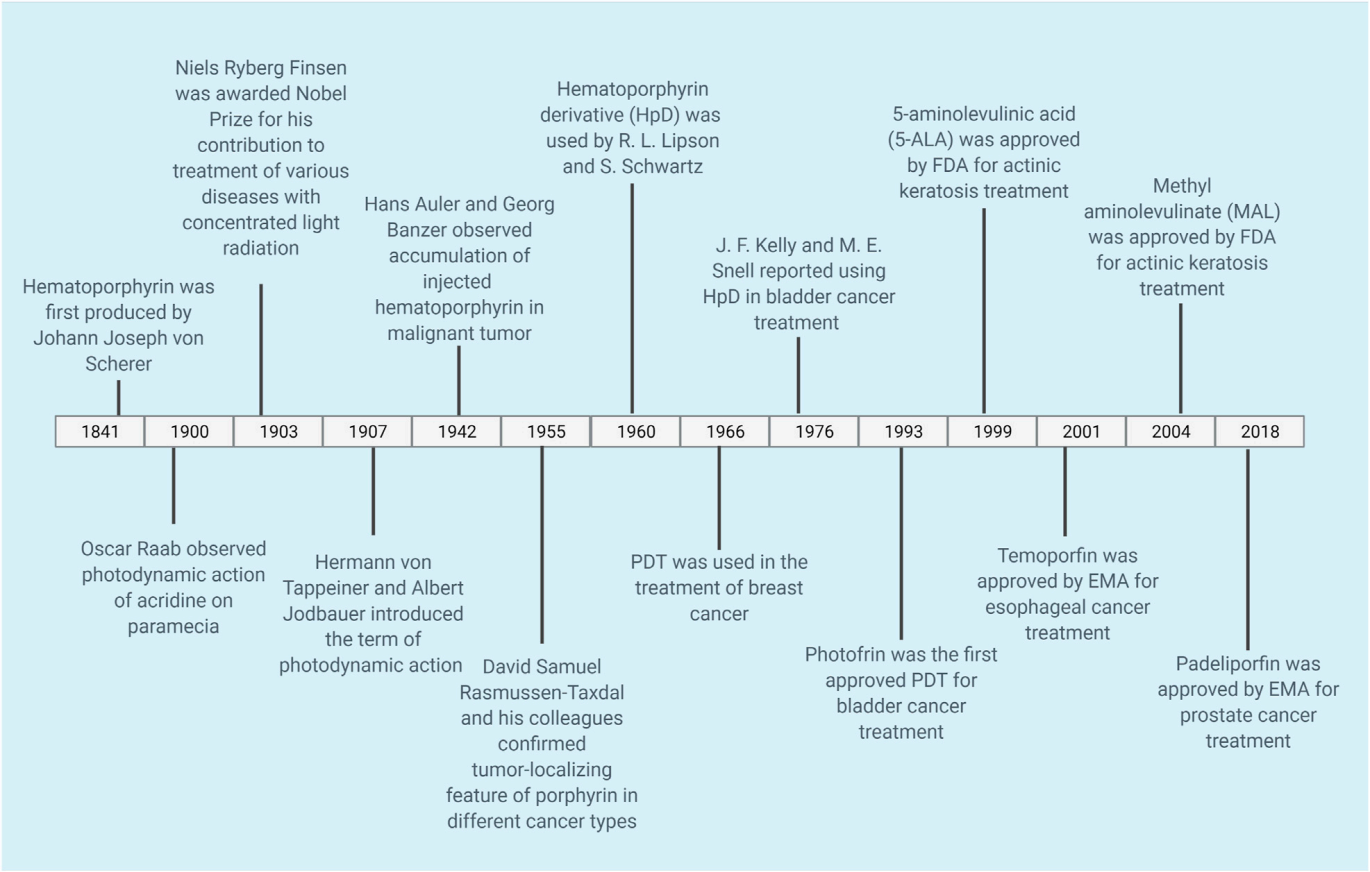
Timeline of PDT. The timeline shows selected applications of PDT for cancer. PDT, photodynamic therapy.

Indeed, PSs have been used as therapeutic agents for more than a century. The timeline of PDT can be seen in Figure 2. The clinical use of eosin in the treatment of skin cancer may be given as one example of the first PDT applications. Tappeiner and Jesionek aimed to treat skin tumors by using topical eosin (Dolmans et al., 2003; Juarranz et al., 2008). PSs are classified into three generations based on their evolution (Allison et al., 2004; Dave et al., 2012; Freitas and Hamblin, 2016; Baskaran et al., 2018). Table 1 demonstrates examples of first, second and third generation PSs.

**TABLE 1:** Selected examples of first, second and third generation PSs. PS, photosensitizer.

First generation PSs	Second generation PSs	Third generation PSs
Porphyrin	Benzoporphyrin	Antibody conjugates
Hematoporphyrin	5-Aminolevulinic acid	Encapsulated agents into carriers (*e.g.* liposomes, micelles, nanoparticles)
	Protoporphyrin IX	
	Phthalocyanine	
	Chlorin	

**FIGURE 3:**
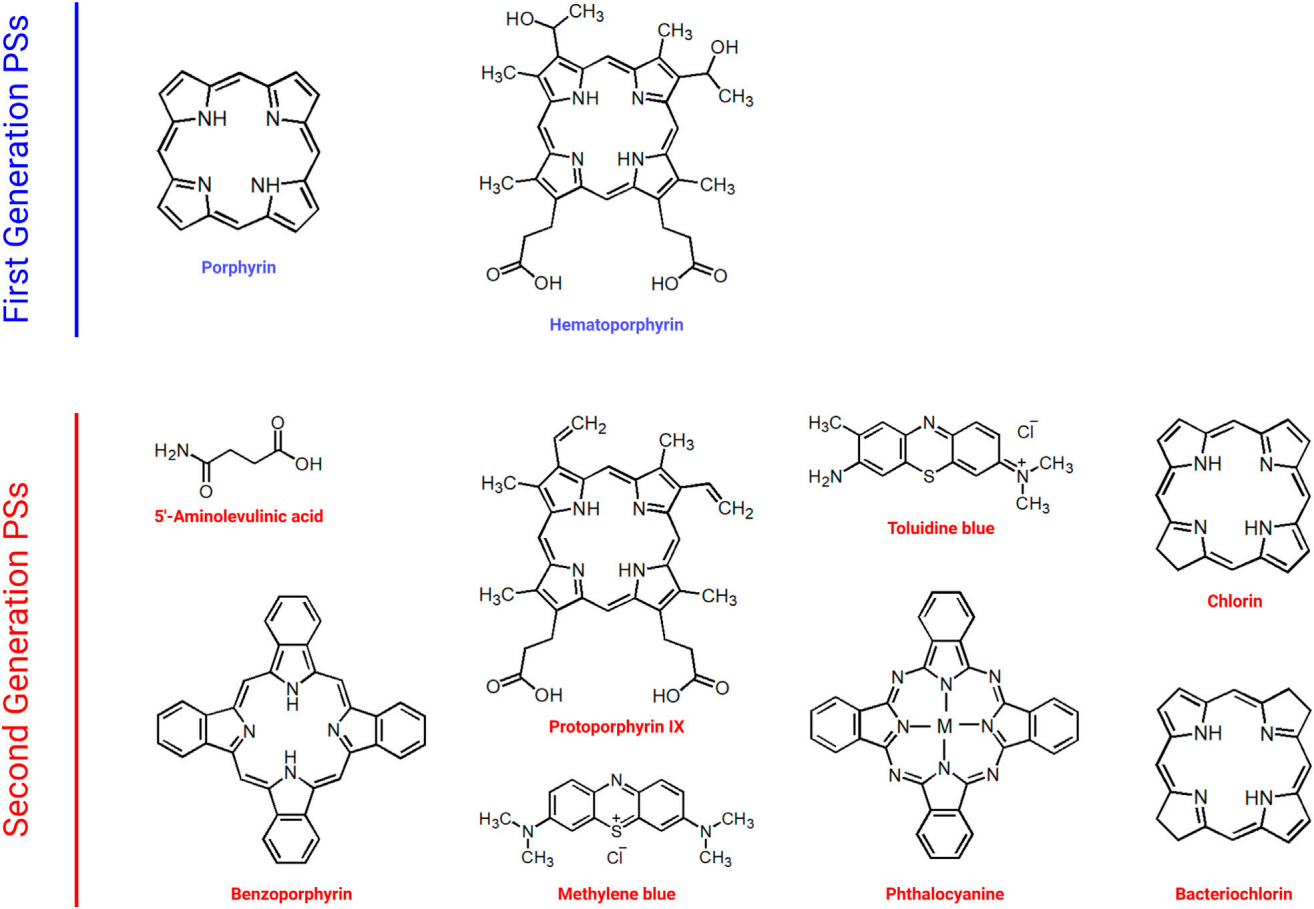
Selected Examples of First and Second Generation PSs. PS, photosensitizer.

Naturally occurring porphyrins and their derivatives constitute the first generation of PSs (Peterson et al., 1992; Schmidt-Erfurth et al., 1998; Clarke et al., 2006; Qiu et al., 2017). Figure 3 demonstrates examples of first and second generation PSs. Hematoporphyrin, which was used in cancer treatment in the 1950s, and Photofrin, which was approved by the FDA in the treatment of various cancers such as lung, esophagus, and cervix, are among the examples of first generation PSs. Indeed, most first generation PSs were developed around 1970s (Freitas and Hamblin, 2016). The first generation PSs were used in early clinical trials of PDT (Spikes, 1990; Kou et al., 2017). Even though first generation PSs were thought to demonstrate favorable photodynamic activity, they bear important disadvantages such as dark cytotoxicity, cutaneous phototoxicity and low absorption bands at red wavelengths as well as issues related with hydrophobicity.

Second generation PSs were developed in order to overcome the problems associated with first generation PSs (Figure 3) (Gomer, 1991). Second generation PSs include 5-aminolevulinic acid, benzoporphyrin, chlorin and phthalocyanine (Allison et al., 2004; Bazylinska et al., 2012; Duchi et al., 2013). In general, second generation PSs are activated with wavelengths above 650 nm and they demonstrate less phototoxicity (Freitas and Hamblin, 2016). In addition, the clearance of second generation PSs from normal tissues is quicker than that of porphyrins (Bazylinska et al., 2012). Second generation PSs also demonstrate high singlet oxygen quantum yield and high solubility in water. Although they can be used in various types of cancer, they might still cause tissue damage due to high toxicity as well as demonstrating low stability. In order to achieve more accurate targeting, various chemical modifications can be made (Nishiyama et al., 2009; Senge, 2012). Such approaches resulted in the generation next generation PSs. Indeed development of specific carriers for the delivery of PSs aiming to cause less phototoxicity in normal tissues may help to improve PDT outcomes.

Third generation PSs utilize available drugs by modifying them (Allison et al., 2004; Josefsen and Boyle, 2008; Mfouo-Tynga et al., 2021). Third generation PSs usually consist of second generation PSs that were modified with specific agents (*e.g.*, antibody conjugation) or were encapsulated into carriers in order to increase the accumulation at the target area (Moser, 1998; Hudson et al., 2005; Staneloudi et al., 2007; O’Connor et al., 2009; Narumi et al., 2016; Nishie et al., 2016; Mfouo-Tynga et al., 2021). Indeed, current studies aim to develop third generation PSs in order to decrease off-target effects and to improve pharmacokinetic properties. An important concept in terms of third generation PSs is the utilization of molecular carriers such as nanoparticles. Such carriers are mainly utilized in order to deliver various PSs to the cells, given the probable poor water-solubility of the PSs (Chilakamarthi and Giribabu, 2017; Kwiatkowski et al., 2018). Various carrier molecules are developed in order to increase the effectiveness of PDT such as liposome, micelle, quantum dot, dendrimer, polymer; magnetic gold, and carbon-based nanoparticles (Abrahamse et al., 2017). For instance, chlorin E6 was incorporated into nanoparticles via formation of ion complexes in order to increase absorption by the tumor (Lee et al., 2013).

Encapsulation of drugs into delivery systems has been as area of extensive research over the last years (Rout et al., 2017; Barras et al., 2018; Fasiku et al., 2021; Karges et al., 2021; Mitchell et al., 2021). Although bioconjugation and encapsulation with targeting moieties seem to be critical approaches in order to develop more effective and specific PSs, it should also be borne in mind that an ideal PS should achieve high quantum yield, display long-wave absorption, cause low dark toxicity, demonstrate favorable pharmacokinetic properties, have high purity and stability (Wöhrle et al., 1998). Furthermore, becoming activated at different wavelengths and having amphiphilic properties can also be considered among the favorable features for PSs (Allison, 2014). Last but not least, it should be kept in mind that classifying drugs into generations does not necessarily mean that all newer drugs are better that the older ones (Allison et al., 2004). Future clinical studies comparing the effectiveness as well as adverse effects of different PSs in large cohorts of patients will pave the way for the development of more potent PSs.

## Clinical Applications of Photodynamic Therapy in Cancer

**FIGURE 4:**
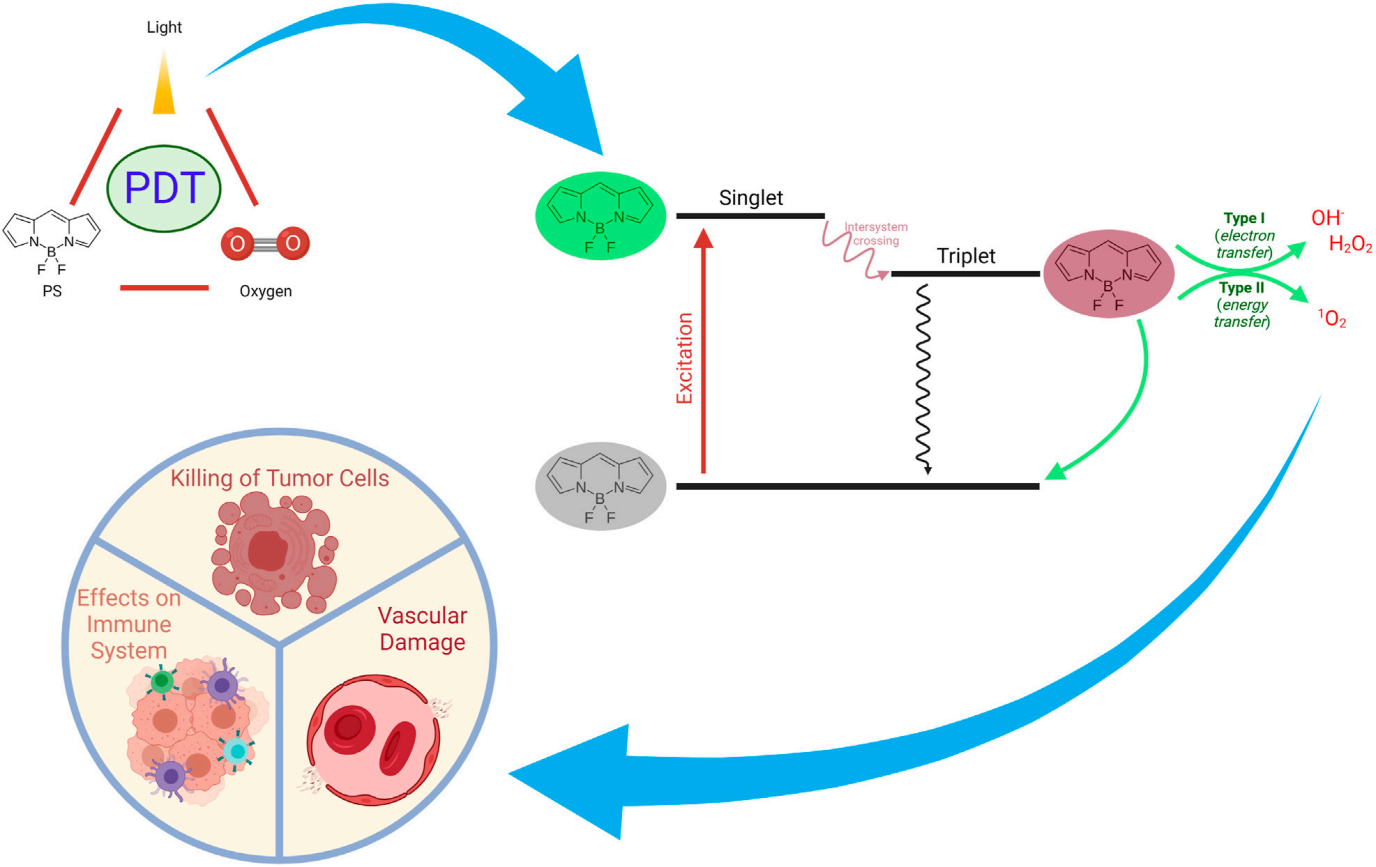
Mechanisms of Action of PDT on Tumors. PDT can exert a plethora of actions such as inducing immune responses, direct killing of tumor cells, and damaging vascular structures.

**FIGURE 5:**
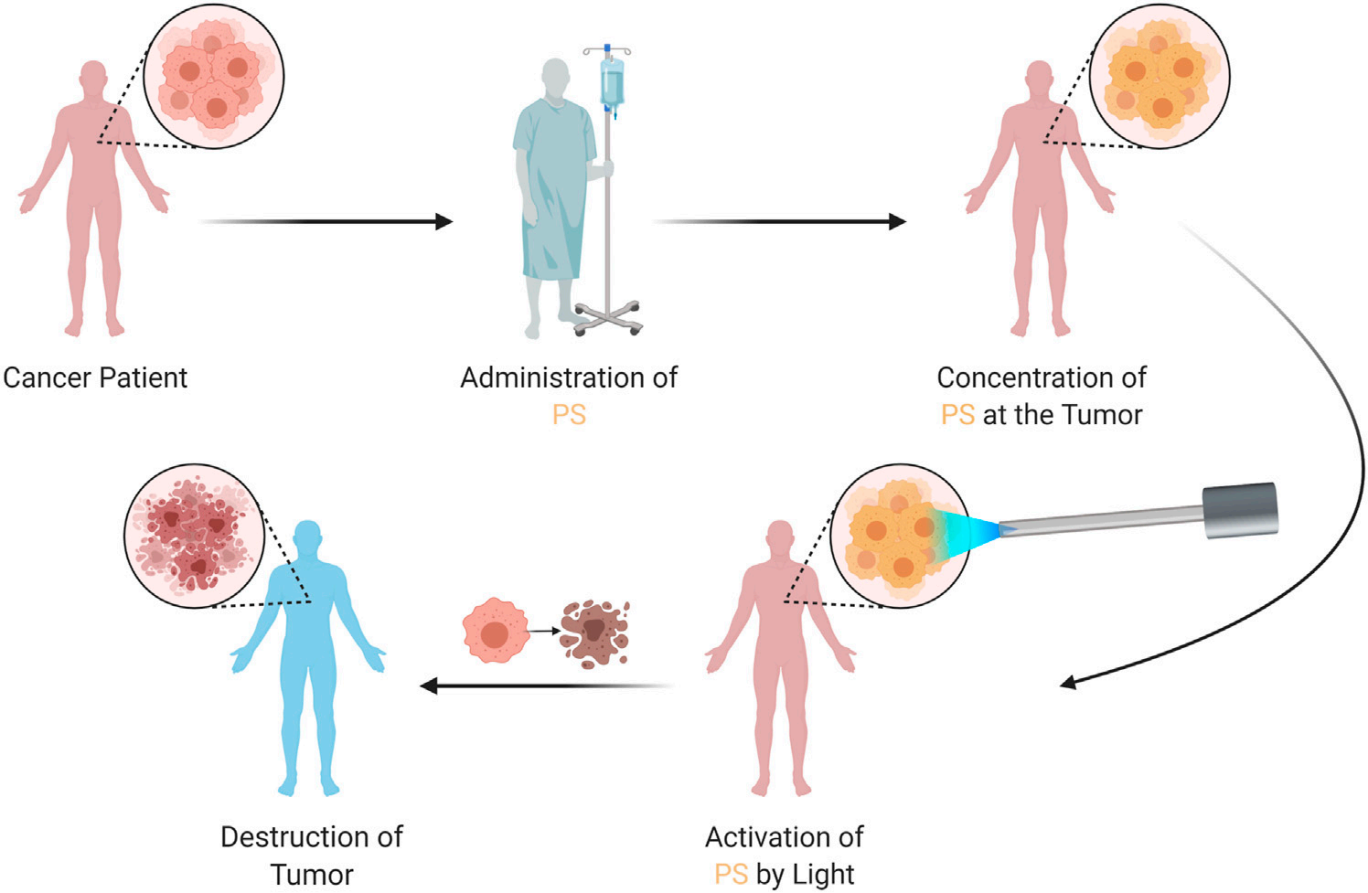
Therapeutic Application of PDT. The patient is administered with the PS, which concentrates at the tumor. The PS is then activated by a specific light, which generates reactive oxygen species; resulting in tumor destruction. PDT, photodynamic therapy; PS, photosensitizer.

PDT is an approach which mainly requires three components, *i.e.* a photosensitizer, light and oxygen (Moore et al., 2009; Allison and Moghissi, 2013b; Dos Santos et al., 2019). Mechanisms of action of PDT on tumors can be seen in Figure 4. In general, PSs are in inactive state unless they are exposed to specific light. Moreover, photodynamic activity is dependent on the presence of oxygen. The light activated PS generates ROS, which mainly result in the effector functions (*e.g.*, killing of tumor cells) (Castano et al., 2006; Avendaño and Menéndez, 2015). PDT comprises two stages of clinical treatment, combining light energy with a PS in order to destroy tumor cells after activation by light (Figure 5 demonstrates therapeutic application of PDT). Several side effects and limitations can restrict the use of some PDT agents (Yoo and Ha, 2012). Problems such as lack of selectivity, poor water-solubility, possible toxic effects on healthy tissues, skin photosensitivity that is associated with certain PSs may prevent the clinical applications of various PDT approaches. Many research groups have undertaken the task to overcome such issues and several promising targeted approaches have been reported over the last years (Turan et al., 2016; Karaman et al., 2019; Ayan et al., 2020). Novel innovative strategies aim to increase the delivery of PSs to tumor tissues, to augment specificity and to increase efficiency (Bugaj, 2011). Further clinical studies and proof of principle approaches will pave the way for better PDT modalities.

### Clinical Efficacy of Photodynamic Therapy in *Cancer*

PDT was the first drug/device combination approved by the FDA (Agostinis et al., 2011). Various PSs have been commercialized or used in clinical trials up to date (Agostinis et al., 2011; Zhao et al., 2021). One of the most frequently used PSs in the clinic is Photofrin (porfimer sodium). The first approval of PDT with Photofrin was obtained in Canada in 1993 for the treatment of bladder cancer (Usuda et al., 2006). In Netherlands and France, Photofrin was approved for the treatment of advanced lung cancers, whereas it was approved in Germany for the treatment of early stage lung cancer (Usuda et al., 2006). In summary, Photofrin has been approved by the FDA for three indications, *i.e.* palliation of patients with esophageal cancer; treatment of microinvasive endobronchial non-small-cell lung cancer (NSCLC) and reduction of obstruction and palliation of symptoms in patients with completely or partially obstructing endobronchial NSCLC; and ablation of high-grade dysplasia in Barrett’s esophagus (Pinnacle Biolgs, 2020). Selected PSs approved by the FDA can be seen in Table 2. It is activated by red light from a laser. Aminolevulinic acid (ALA) has been approved by the FDA for the indication of topical treatment of minimally to moderately thick actinic keratoses of the face or scalp, or actinic keratoses of the upper extremities (in summary) (Table 2) (DUSA, 2018). In addition, oral solution of ALA has been approved by the FDA as an optical imaging agent indicated in patients with glioma as an adjunct for the visualization of malignant tissue during surgery (in summary) (Table 2) (NXDC, 2018). Methyl aminolevulinate has been approved by the FDA for the indication of treatment of thin and moderately thick, non-hyperkeratotic, non-pigmented actinic keratoses of the face and scalp in immunocompetent patients (in summary) (Table 2) (Galderma Labs LP, 2012). Hexaminolevulinate has been approved by the FDA as an optical imaging agent indicated for use in the cystoscopic detection of carcinoma of the bladder (in summary) (Table 2) (Photocure ASA, 2018). Indeed, several preclinical and clinical studies are ongoing in order to develop new PSs and PDT approaches.

**TABLE 2:** Selected PSs approved by the FDA. PS, photosensitizer, NSCLC, non-small-cell lung cancer.

Name of PS	Indications and usage
**Porfimer sodium**	• Palliation of patients with esophageal cancer
	• Treatment of microinvasive endobronchial NSCLC and reduction of obstruction and palliation of symptoms in patients with completely or partially obstructing endobronchial NSCLC
	• Ablation of high-grade dysplasia in Barrett’s esophagus
**Aminolevulinic acid**	• Topical treatment of minimally to moderately thick actinic keratoses of the face or scalp, or actinic keratoses of the upper extremities
	• (Oral solution) as an optical imaging agent indicated in patients with glioma as an adjunct for the visualization of malignant tissue during surgery
**Methyl aminolevulinate**	• Treatment of thin and moderately thick, non-hyperkeratotic, non-pigmented actinic keratoses of the face and scalp in immunocompetent patients
**Hexaminolevulinate**	• An optical imaging agent indicated for use in the cystoscopic detection of carcinoma of the bladder

### Therapeutic Applications of Photodynamic Therapy

**FIGURE 6:**
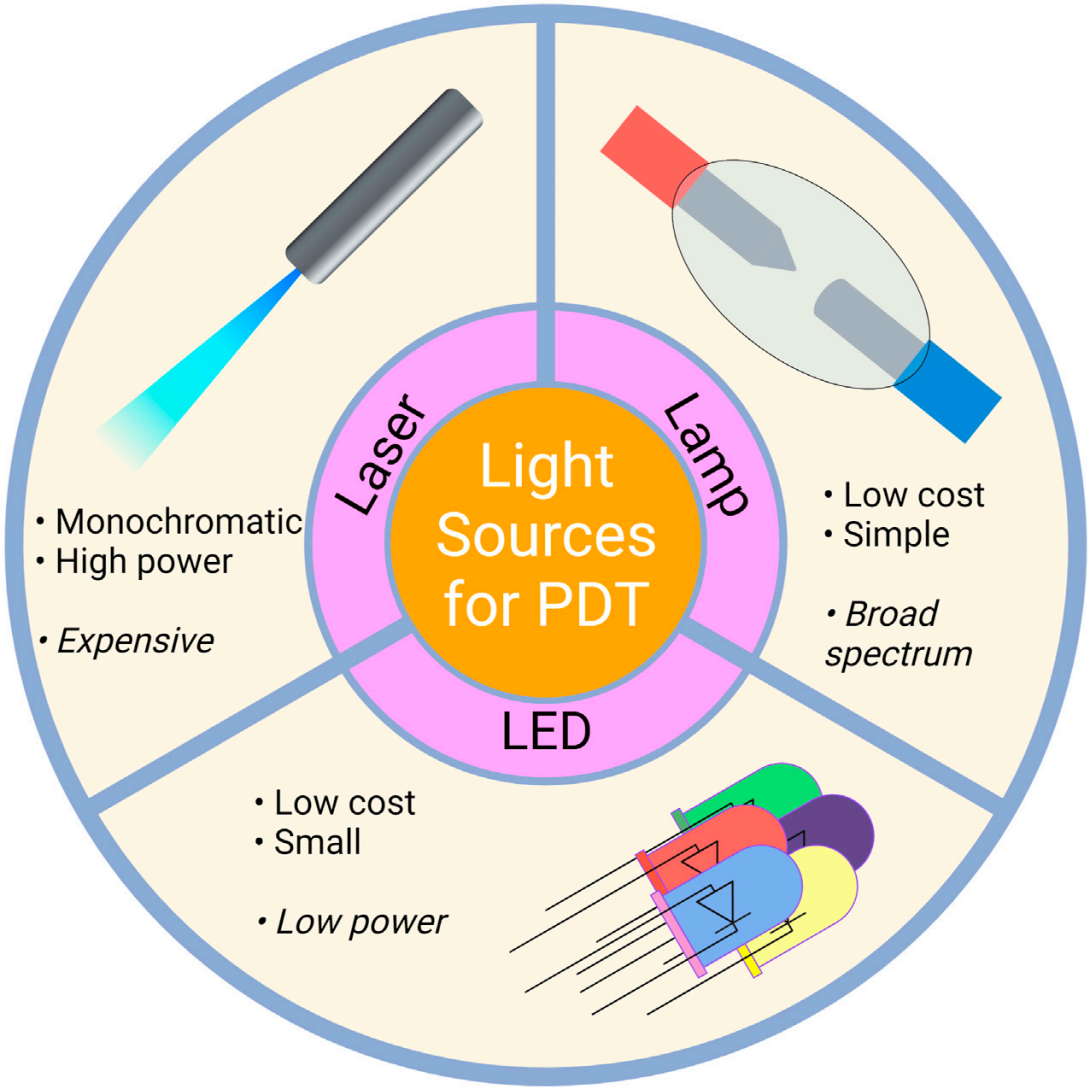
Light Sources Utilized in PDT. Laser, lamp and LED sources can be used for PDT applications. LED, light emitting diodes.

Photodynamic therapy is indeed a treatment method used to treat various diseases utilizing the photosensitizer’s photophysical and chemical properties. Mechanistically, photodynamic treatment consists of two steps. The first step is the excitation of PS, which is induced by light. Two groups of light sources can be utilized in terms of the photoexcitation stage of PDT, *i.e.*, laser and non-laser. Light sources utilized in PDT can be seen in Figure 6. Laser light sources include Argon Lasers and Argon-pumped Dye Lasers, Metal Vapor-pumped Dye Lasers, Solid State Lasers, Optical Parametric Oscillators, and Diode Lasers. On the other hand, Tungsten Filament Quartz Halogen Lamps, Xenon Arc Lamps, Metal Halide Lamps, Phosphor-coated Sodium Lamps, and Fluorescent Lamps are among the non-laser light sources. In addition to these sources, Light Emitting Diodes (LED) and Femtosecond Solid State Lasers may also be utilized (Brancaleon and Moseley, 2002).

The reaction of the photochemically excited PS with molecular oxygen constitutes the second step of PDT. Upon excitation by specific light, one of the electrons of the PS in the ground state (S_0_), which has two electrons in opposite spins in a molecular orbit involving lower energy, is excited towards the molecular orbit involving higher energy (singlet state = S_1_) without changing its spin. According to the Jablonski energy diagram, PS with high energy orbit or singlet state can transform to the ground state in a short time by fluorescent radiation (emission) or heat releasing (internal conversion = IC) (Figure 4). By performing intersystem crossing (ISC) to the triplet state, it may transform into a long-lasting form. The triplet state’s transformation occurs in parallel with the transformation in the singlet state by phosphorescence radiation (emission) or heat releasing (IC). When PDT is applied in biological systems, PS reaches a high-energy triplet state as a result of the absorption of light. Then, PS reacts with cellular substrates (biomolecules) (Type I reaction) and ROS are generated. On the other hand, energy transfer to molecular oxygen (excitation energy transfer, EET) leads to the conversion of oxygen to singlet oxygen (Type II reaction) (Figure 4). In both cases, the reaction of the generated molecules with vital cellular components such as lipids, proteins, and nucleic acids can result in toxicity to the organism (Chilakamarthi and Giribabu, 2017; Kwiatkowski et al., 2018).

Depending on the type of PDT and its ROS production efficiency, PDT may affect cellular antioxidants and heat shock proteins as well as anti-tumor immune responses. Transformation of ROS into nontoxic molecules is catalyzed by antioxidant enzymes inside the cell, such as superoxide dismutase, catalase and glutathione peroxidase. Antioxidant enzymes take active part in the breakdown of superoxide anions and hydrogen peroxides that accumulate in the cell. Additionally, small molecules such as glutathione, vitamins E and C also help to ensure intracellular control of ROS. It has been reported that cancer cells generate more ROS than normal cells. Therefore, cancer cells require a robust antioxidant system in order to be able to survive. It was reported that the cytotoxic effects of PDT are enhanced when antioxidant enzymes or glutathione synthesis mechanisms are targeted during PDT (Ayan et al., 2020). It was demonstrated that l-buthionine sulfoximine, which is a glutathione synthesis inhibitor, boosts PDT effects. Similarly, targeting cyclooxygenase, which regulates inflammation and homeostasis, and heat shock proteins, which are activated by oxidative stress and have an essential functional role in protein folding, can increase the effectiveness of PDT (Kimani et al., 2012; Dabrowski and Arnaut, 2015). PDT might achieve direct killing of tumor cells by generating ROS or singlet oxygen in the tumor cells. Moreover, it can also result in tumor associated vascular damage and; hence, tumor infarction (Castano et al., 2005). Finally yet importantly, PDT may also induce enhanced immune responses against the tumor (Dolmans et al., 2003).

PDT generally demonstrates fewer side effects and less toxicity than chemotherapy and/or radiotherapy used in conventional cancer treatment (Li X. et al., 2020). Indeed, PDT has been used in cancer treatment for some time. More than two hundred clinical trials have been conducted until recently. In addition, there are currently numerous ongoing studies. Selected studies can be seen in Table 3. In a porfimer sodium based PDT study conducted with 1,440 patients diagnosed with basal cell carcinoma, which is the most common skin cancer type, PDT was reported to demonstrate an initial complete response rate of 92%, with a recurrence rate of less than 10% at 4 years (Zeitouni et al., 2001; Garcia-Zuazaga et al., 2005; Agostinis et al., 2011). In addition to cancer, PDT may also be used in other diseases such as those of the skin and the eye (Wilson and Patterson, 2008). It is also an effective treatment option for Barrett’s esophagus, unresectable cholangiocarcinoma and non-melanoma skin lesions.

**TABLE 3:** Selected recent active PDT studies for malignant diseases obtained from ClinicalTrials.gov database.

Title	Status	*Cancer* type	Photosensitizer	Excitation wavelength	Phase	Start date	ClinicalTrials.gov identifier
Study to evaluate the safety and efficacy of BF-200 ALA (Ameluz^®^) and BF-RhodoLED^®^ in the treatment of superficial basal cell carcinoma (sBCC) with photodynamic therapy (PDT)	Recruiting	Basal cell carcinoma	ALA-PDT (Ameluz®-PDT)	635 nm	Phase 3	September 25, 2018	NCT03573401
Use of jet-injection in photodynamic therapy for basal cell carcinoma	Recruiting	Basal cell carcinoma	Jet-injection (AirGent2.0) of ALA (levulan kerastick)	400–450 nm	Phase 2	September 9, 2020	NCT04552990
Intravesical photodynamic therapy (PDT) in BCG refractory/Intolerant non-muscle invasive bladder cancer (NMIBC) patients	Recruiting	Bladder cancer	TLD-1433 (Ru(II) polypyridyl complex)	520–540 nm	Phase 2	August 30, 2019	NCT03945162
PCI treatment/Gemcitabine & chemotherapy vs chemotherapy alone in patients with inoperable extrahepatic bile duct cancer	Recruiting	Cholangiocarcinoma	Fimaporfin	435 nm	Phase 2	May 23, 2019	NCT04099888
A trial of a new type of photodynamic therapy (VTP) in the treatment of patients with cancer of the esophagus who have trouble swallowing	Recruiting	Esophagogastric cancer	WST11-mediated vascular-targeted photodynamic therapy (VTP)	753 nm	Phase 1	April 26, 2017	NCT03133650
Intraoperative photodynamic therapy of glioblastoma	Active, not recruiting	Glioblastoma	Gliolan^®^ (5-ALA- PpIX (protoporhyrin IX))	400–410 nm	Not applicable	May 5, 2017	NCT03048240
Stereotactical photodynamic therapy with 5-aminolevulinic acid (Gliolan^®^) in recurrent glioblastoma	Recruiting	Glioblastoma	Gliolan^®^ (5-ALA- PpIX (protoporhyrin IX))	400–410 nm	Phase 2	March 2021	NCT04469699
Porfimer sodium interstitial photodynamic therapy with or without standard of care chemotherapy in treating patients with locally advanced or recurrent head and neck cancer	Recruiting	Head and neck carcinoma	Porfimer sodium (Photofrin^®^)	630 nm	Phase 2	January 16, 2019	NCT03727061
Endobronchial ultrasound guided interstitial photodynamic therapy in treating patients with locally advanced lung cancer	Recruiting	Lung carcinoma	Porfimer sodium (Photofrin^®^)	630 nm	Early phase 1	February 4, 2020	NCT03735095
Photodynamic therapy for prevention of nonmelanoma skin cancer in organ transplant recipients	Recruiting	Nonmelanoma skin cancers	ALA (levulan kerastick)	400–450 nm	Not applicable	February 2016	NCT02751151
Photodynamic therapy with HPPH compared to standard of care surgery in treating patients with oral cavity cancer	Active, not recruiting	Oral cavity squamous cell carcinoma	HPPH (photochlor) (2–1 [heyloxyethyl]-2-devinylpyropheophorbide-a)	665 nm	Phase 2	March 30, 2017	NCT03090412
Ultrasound-guided verteporfin photodynamic therapy for the treatment of unresectable solid pancreatic tumors or advanced pancreatic cancer, VERTPAC-02 study	Recruiting	Pancreatic carcinoma	Verteporfin	690 nm	Phase 2	December 6, 2016	NCT03033225
Study of the efficacy, safety and quality of life after TOOKAD^®^ soluble (VTP) for intermediate risk prostate cancer	Active, not recruiting	Prostate cancer	TOOKAD^®^ soluble VTP	753 nm	Phase 2	October 2, 2017	NCT03315754
Study of erectile dysfunction, urinary incontinence and related QoL after TOOKAD^®^ VTP for low risk prostate cancer	Active, not recruiting	Prostate cancer	TOOKAD^®^ soluble VTP	753 nm	Phase 4	January 21, 2019	NCT03849365
Clinical study to assess the safety and adequacy of effectiveness of the SpectraCure P18 system	Recruiting	Prostate cancer	Verteporfin	690 nm	Phase 1	March 21, 2017	NCT03067051
HS-201, an HSP90 inhibitor-linked verteporfin for detection of solid malignancies	Recruiting	Solid tumor	HS-201 (verteporfin-tethered HSP90 inhibitor)	690 nm	Phase 1	July 15, 2020	NCT03906643
Phase I study in advanced malignancies	Recruiting	Solid tumor	ALA (5-aminolevulinic acid)	635 nm	Phase 1	July 23, 2020	NCT04381806
Treatment of tumors in the urinary collecting system of the kidney or ureter using a light activated drug (WST11)	Recruiting	Upper tract urothelial carcinoma	WST11	753 nm	Phase 1	August 1, 2018	NCT03617003

#### Targeting Strategies in Photodynamic Therapy

Tumor-targeted PDT can be divided into two groups, *i.e.*, passive targeting and active targeting (Pernot et al., 2013). Passive targeting takes advantage of physiological and morphological differences between normal and tumor tissues to achieve tumor-selective targeting as well as PS accumulation. The uncontrolled proliferation of tumor cells results in vessels with irregular structure. Tumor vessels are usually leaky and tortuous with irregular branching in contrast to normal tissues (Carmeliet and Jain, 2011). Due to the heterogeneous structure of the vessels as well as the basement membrane and irregular endothelial cells, most drugs that are administered accumulate in tumor tissues. In addition, components of the extracellular matrix (ECM) such as collagen, elastin and hyaluronan are generally expressed more in tumor tissues than their normal counterparts. Although such conditions can be regarded as an obstacle for the transfer of some drugs, it is known that porphyrin derivatives interact with collagen. Semi-specific passive targeting may be achieved via using such characteristics of the tumor.

Various surface receptors are expressed more by tumor cells than normal cells. Such receptors can be utilized in terms of active targeting (Schmitt and Juillerat-Jeanneret, 2012). For instance, HER2 is overexpressed in 15–30% of invasive breast cancers (Burstein, 2005; Iqbal and Iqbal, 2014). HER2, which is also known as ErbB2, is a member of the HER receptor family (Yu et al., 2018). Breast cancers may bear up to 25–50 copies of the HER2 gene, resulting in an estimated number of two million receptors expressed at the tumor cell surface (Kallioniemi et al., 1992), while a normal cell surface has much less HER2 receptors. Such a difference in receptor expression underlies an active targeting strategy, which takes advantage of the combination of PDT with trastuzumab. Indeed, trastuzumab, which is a humanized anti-HER2 antibody, is the first HER2-targeted therapeutic monoclonal antibody approved by the FDA in 1998 for the therapy of metastatic HER2^+^ breast cancer (Rossi et al., 2016; Shu et al., 2020). In addition to targeting receptors with specific monoclonal antibodies, conjugation of PSs with specific ligands that bind to the receptors is also a promising approach. Mannose conjugated PSs (Zhang et al., 2017) to target mannose receptors that may be overexpressed in breast cancer cells, EGF conjugated PSs for targeting EGFR receptors (Kuo et al., 2010), cannabinoid CB2 receptor (a G-protein coupled receptor)/translocator protein (a mitochondria membrane receptor) targeted PSs (Yang et al., 2017; Zhang J. et al., 2018) can also be utilized for active targeting strategies in breast cancer (Savellano et al., 2005; Eccles, 2011; Shirasu et al., 2013).

It is widely known that the tumor *milieu* contains various types of cells in addition to tumor cells (*e.g.*, immune cells and fibroblasts) (Gunaydin et al., 2015; Gok Yavuz et al., 2019; Jin and Jin, 2020; Gunaydin, 2021). The efficacy of PDT may be affected by the components of the complex tumor microenvironment. Thus, recent approaches incorporating nanotechnology strategies in order to augment the effects of PDT via remodeling the tumor microenvironment (*e.g.*, reshaping tumor vessels, ECM, and anti-tumor immune responses) are exciting (Liang et al., 2020; Sorrin et al., 2020). Moreover, increased selectivity for cancer cells may be achieved by altering PSs with bioresponsive elements. Targeting overexpressed enzymes in tumor cells in contrast to normal cells is an encouraging approach in order to generate ROS specifically in tumor cells (Gunaydin et al., 2021).

In normal healthy cells, glucose taken into the cell under normoxic conditions is broken down into pyruvate in the cytosol. The pyruvate is transported to the mitochondria, where it is oxidized by pyruvate dehydrogenase. This is followed by the citric acid cycle. Under anoxic or hypoxic (low partial oxygen pressure) conditions, the reduction of pyruvate to lactate is catalyzed by lactate dehydrogenase. Pyruvate cannot be transported to the mitochondria, with the inhibition of pyruvate dehydrogenase by pyruvate dehydrogenase kinase (PDK). Indeed, hypoxia is a common feature of the tumor microenvironment (Gunaydin and Gedik, 2019). While approximately 38 ATP molecules are generated as a result of each cycle in healthy aerobic cells, 2 ATP molecules are generated as a result of each cycle in the cancer cells. *Cancer* cells maintain energy production by increasing the expression of glucose transporter (GLUT) on the cell surface and; thus, glucose uptake into the cell. It is known that cancer cells prefer aerobic glycolysis under normoxic (typical oxygenated environment) conditions. This mechanism is called the Warburg effect (Gatenby and Gillies, 2004; DeBerardinis et al., 2008; Gogvadze et al., 2008; Vander Heiden et al., 2009). Thus, novel PDT approaches can also be based on the Warburg effect. Kataoka *et al.* reported developing a third generation PDT by synthesizing a sugar conjugated chlorin PS, with enhanced cancer cell selective accumulation, since tumors cells consume higher levels of glucose than normal cells due to the Warburg effect (Kataoka et al., 2017). They reported that glucose conjugated chlorin based PDT demonstrated more potent anti-tumor effects than second generation talaporfin mediated PDT. Moreover, PDT with glucose conjugated chlorin was found to induce immunogenic cell death (Kataoka et al., 2017). In another study, Gan *et al.* reported that pyruvate kinase M2, which is a rate-limiting enzyme of glycolysis, was downregulated and glucose uptake was inhibited in cells that were exposed to PDT with 5-aminolevulinic acid (5-ALA) at 4 h after treatment. Interestingly, they reported important increases in PKM2 expression and glucose uptake at 24 h after PDT (Gan et al., 2020). Such findings suggest that PDT may indeed drive the Warburg effect in a time dependent manner.

5-ALA is a frequently used PDT agent, due to its high endogenous accumulation. It is the first compound in the porphyrin synthesis pathway that results in chlorophyll in plants and heme in mammals (Chilakamarthi and Giribabu, 2017). 5-ALA, which is synthesized under physiological conditions from succinyl coenzyme A and glycine in mitochondria, leads to the biosynthesis of protoporphyrin IX (PpIX), the last precursor of heme in mitochondria. PpIX is then catalyzed by ferrochelatase, which produces hemoprotein (heme) by reducing the iron (III) ion to iron (II). At the end of this process, heme is produced. Due to the Warburg effect seen in cancer cells, the enzyme ferrochelatase becomes inactive. This leads to the increased accumulation of PpIX in cancer cells as a result of the utilization of exogenous 5-ALA. As a result of PDT via the photoactive feature of PpIX; ROS and singlet oxygen as well as superoxides increase in the cell, which in turn result in cellular death. In addition to PDT applications with 5-ALA and hexaminolevulinate, which is used as an optical imaging agent in the cystoscopic detection of carcinoma of the bladder, monoclonal antibody conjugated PDT and photoimmunotherapy (PIT) strategies are currently being developed (Inoue, 2017; Railkar and Agarwal, 2018).

#### Limitations of Photodynamic Therapy

PDT is generally ineffective for large or deep-seated tumors due to the limited penetration depth of light in biological tissues. One of the major limitations of PDT is that it cannot be applied to the whole body in advanced stage cancer. For this reason, its utilization is limited to the treatment of precancerous lesions and regional malignancies (Brown et al., 2004; Li X. et al., 2020). Difficulty in treating large tumor masses is another issue in terms of clinical PDT. Moreover, singlet oxygen, which is generated as a result of PDT, has a very short half-life. Thus, the effects of PDT are mainly limited to the area of photosensitization (Moan and Berg, 1991). Given the fact that PDT is highly dependent on the presence of oxygen in the tissues, tumor hypoxia can significantly hamper the effectiveness of PDT (Bhandari et al., 2019; Shen et al., 2021). Indeed, depletion of oxygen due to PDT itself might also decrease PDT efficiency. In addition, problems related with skin toxicity should also be taken into account in terms of clinical PDT applications (Borgia et al., 2018).

The period of time between the administration of the drug and the application of light is called the *drug-light interval*. Optimum drug-light intervals are mostly assumed to be the times at which there is a maximum differential in PS retention between the tumor and normal tissue (Cramers et al., 2003). However, optimum drug-light interval might vary from patient to patient or lesion to lesion (Wang et al., 2021). Such variations render the application of standardized protocols difficult. It should also be borne in mind that tumor destruction via targeting the vascular structures is also an important part of clinical PDT. Thus, plasma levels of the PSs as well as the exposure of endothelial cells to the PSs may prove to be crucial factors for effective PDT. Indeed, Li and Luo demonstrated that the anti-tumor effects of Photofrin PDT were achieved mostly by the destruction of tumor blood vessels at short drug-light intervals. On the other hand, tumor cells were destroyed directly by PDT mediated cytotoxicity at long drug-light intervals (Li and Luo, 2009). In general, PSs that directly destruct the target cells should have a relatively longer drug-light interval. On the other hand, PSs with a short drug-light interval may be targeted to tumor related vascular structures rather than the tumor cells in order to improve the effectiveness of treatment (Allison and Moghissi, 2013a).

Moreover, the depth of PDT application in cancer treatment is limited, given the fact that light cannot penetrate beyond a few millimeters of tissue (Stolik et al., 2000; Gunaydin et al., 2021). The treatment efficiency decreases in deep-seated and spreading tumors due to the low tissue penetration of light. The deep-PDT strategy, which was developed with new photo converting nanoparticles and/or NIR light/X-ray/self-luminescence excitation methods, aims to overcome this limitation (Fan et al., 2016; Li et al., 2021). One approach takes advantage of the two-photon excitation technique, which involves absorption of two photons in order to reach an excited state (Bolze et al., 2017). This technique, which allows for the activation of PSs with two-photon absorption, may aid in improving light penetration depth (Lan et al., 2017).

### Assessment of Photodynamic Therapy Therapeutic Efficacy in the Clinic

It is of utmost importance to identify early predictors in order to determine the response to treatment. Huang *et al.* reported that gadolinium contrast-enhanced magnetic resonance imaging (MRI) is superior to diffusion weighted images at 7 days after PDT (Huang et al., 2006). The MRI results correlated with the percentage of necrosis in a canine model (Huang et al., 2006). On the other hand, Haider *et al.* demonstrated that contrast-enhanced MRI showed irregular margins of intra-prostatic treatment effect and suggested that tissue sensitivities to vascular targeted PDT with palladium-bacteriopheophorbide varied (Haider et al., 2007). Sirotkina *et al.* utilized optical coherence angiography to monitor treatment response following vascular targeted PDT (Sirotkina et al., 2019). In another study, Gross *et al.* investigated the utilization of blood oxygenation level-dependent contrast MRI in order to monitor real-time efficacy of PDT (Gross et al., 2003). Such an approach might be useful in monitoring PDT treatment. Bioluminescence imaging was also investigated as a means of success rate assessment 24 h after vascular targeted PDT (Fleshker et al., 2008). Luciferase transfected (luminescent) tumor cells allowed for the imaging of the tumor before and 24 h after PDT. PDT treatment response was assessed based on the presence of bioluminescence imaging signal. Using the treatment response information, researchers treated the mice that failed the first treatment again; thus, reaching a cumulative treatment success rate of 90% from 75% (Fleshker et al., 2008). The utilization of bioluminescence imaging in *in vivo* animal models may assist in determining response to PDT. Ultrasonography, which is cheaper than MRI, may also prove to be useful in terms of assessing PDT efficacy and it may improve PDT mediated outcomes in cancer. Ultrasonography can be implicated in tracking the uptake of PS, destruction of vessels and evaluating the overall tumor responses (Hester et al., 2020). The tissue distribution of the PS can also be evaluated by taking biopsy samples and analyzing the fluorescence (Moore et al., 2009). Furthermore, strategies to detect intratumoral drug, light and oxygen in order to monitor treatment have also been investigated. In addition to such strategies, computer modelling of PDT can also be helpful, as such approaches may assist PDT in the clinical setting (Jankun et al., 2005).

### Photodynamic Diagnosis

Several types of PSs can be used for diagnostic purposes in terms of tumors (Beharry, 2018). Such approaches are generally called photodynamic diagnosis (PDD). As its name implies, PDD utilizes fluorescent PS agents in order to identify tumor tissues. In PDD, a PS which is selective for the target tumor cells is utilized (Dobson et al., 2018; Kim and Wilson, 2020; Nompumelelo Simelane et al., 2020; Fukuhara et al., 2021; Luan et al., 2021; Owari et al., 2021). The PS can be excited with a light source at a specific wavelength. The emitted light enables identification of the tumor cells. PDD can be regarded as part of fluorescent guided resection, which is a kind of Image Guided Surgery (Allison, 2016). This approach has the potential to improve clinical outcome. PDD can turn into PDT via increasing the intensity/duration of photoirradiation (Dobson et al., 2018). It should be noted that ROS that are generated in PDT are able to harm the PS, causing the PS to become non-fluorescent. ALA seems to have a potential for PDD in various tumors, due to its ability to discriminate neoplastic tissues from normal tissues (Nokes et al., 2013). ALA fluorescence microscopy has been proposed to be a specific biological tumor marker for malignant glioma resection (Hefti et al., 2010). It may assist in discriminating tumor tissue from normal brain tissue. Stummer *et al.* reported that ALA enabled more complete resections of contrast-enhancing tumor, resulting in improved progression free survival in patients with malignant glioma (Stummer et al., 2006). Moreover, PDD was reported to detect more bladder tumor-positive patients, especially more with carcinoma *in situ*, than white-light cystoscopy (Kausch et al., 2010). Kausch *et al.* concluded that a longer recurrence free survival was achieved and more patients had a complete resection when diagnosed with PDD (Kausch et al., 2010). Similarly, Mowatt *et al.* reported that PDD identified more bladder tumors than white-light cystoscopy. PDD with ALA enabled a more complete approach at transurethral resection of bladder tumor and improved recurrence-free survival (Mowatt et al., 2011). Turan *et al.* demonstrated a concept of molecular demultiplexer, which is able to autonomously switch modes from PDT to PDD when apoptosis is induced (Turan et al., 2018). Such an intelligent molecular automaton has shown a way of moving ahead to meet the challenge of confinement of unintended damage by excessive ^1^O_2_ production. It is clear that early detection of a tumor is very important in terms of improving survival rate. Furthermore, combination of PDD and PDT may prove to be very efficient. Near infrared fluorescence imaging is most likely to improve the concept of tumor targeted imaging, due to the features such as low tissue auto-fluorescence and high tissue penetration depth of near infrared spectrum (Luo et al., 2011).

## Photodynamic Therapy Combined With Other Therapy Modalities

Combination of PDT with other therapeutic modalities seems to be promising in terms of enhancing effectiveness against tumor (Firczuk et al., 2011). Several features of PDT such as minimal systemic effects and low long term morbidity render it a suitable option for combination therapy approaches (Agostinis et al., 2011). PDT has been proposed to be effectively combined with other anti-cancer therapeutic modalities, as the mechanism of action of PDT is unique (Juzeniene et al., 2007). Given the fact that the targets of radiotherapy, chemotherapy and PDT differ; combination of PDT with such treatments may yield better results than by single treatments (Juzeniene et al., 2007). Moreover, the utilization of lower doses in a combination therapy setting may cause less side effects and better results than single therapy (Yoo and Ha, 2012). In a study by Gupta *et al.*, PDT with the use of intratumoral administration of specific antibodies conjugated to PSs was proposed to significantly reduce the toxicity to normal tissues (Gupta et al., 2004). Conjugating PSs with monoclonal antibodies that are specific for antigens on tumor cells may allow for the targeting of tumors (Kwitniewski et al., 2008). Bai *et al.* proposed that gene therapy and PDT could be combined for treating nasopharyngeal carcinoma (Bai et al., 2011).

### PDT Combined With Chemotherapy

Nahabedian *et al.* demonstrated that combination of PDT with chemotherapy can yield increased tumoricidal effects (Nahabedian et al., 1988). Several types of tumors were reported to display resistance to platinum analogues and platinum nanomaterials were suggested as possible alternatives for anti-tumor treatment (Chien et al., 2013; Davis et al., 2014; Pedone et al., 2017; Yin et al., 2017; Loveday et al., 2020; Esim et al., 2021). Wang *et al.* reported that combined chemotherapy and PDT was effective in terms of killing cisplatin resistant tumor cells (Wang et al., 2015). Similarly, Antoni *et al.* also reported an additive effect of cisplatin and a zinc porphyrin on human ovarian cancer cells (Antoni et al., 2015). Moreover, PDT might be implicated in attenuating drug resistance. Multidrug resistance (MDR) is a critical mechanism, which can cause tumor cells to become resistant to chemotherapeutic drugs (Persidis, 1999). The development of MDR to chemotherapy may constitute an important issue in terms of the treatment of tumors (Gillet and Gottesman, 2010). Several mechanisms have been found to be associated with resistance to chemotherapy such as cellular pumps dependent, increased metabolism of drugs, decreased drug entry and defective apoptotic pathways (Majidinia et al., 2020). Hence, targeting MDR via chemosensitizers combined with anti-tumor compounds is an interesting approach (Avendano and Menendez, 2002). In line with such studies, PDT is able to attenuate multidrug resistance via decreasing drug efflux by lowering P-glycoprotein level (Shi et al., 2017; Zhao et al., 2018). Thus, combination of PDT and chemotherapy may be useful in terms of preventing drug resistance (Li X. et al., 2020). Jin *et al.* reported that combination of PDT and chemotherapy may prove to be effective against cardiac cancer (Jin et al., 1992). In another study, the combined chemotherapy and PDT with N-(2-hydroxypropyl) methacrylamide copolymer-bound anti-cancer drugs was shown to be effective against human ovarian carcinoma in nude mice (Peterson et al., 1996). Ma *et al.* studied the combination of PDT with meso-tetra (di-adjacent-sulphonatophenyl) porphine and vincristine or taxol in a mouse mammary tumor model (Ma et al., 1996). They found an increased anti-tumor effect when vincristine was administered 6 h before PDT. In addition, they also reported that the anti-tumor activity of PDT could be enhanced when taxol was utilized 6 h prior to PDT or immediately after or before PDT (Ma et al., 1996). Khdair *et al.* analyzed the anti-cancer efficacy of doxorubicin in combination with methylene blue mediated PDT in a mouse mammary adenocarcinoma tumor model (Khdair et al., 2010). The results showed that nanoparticle mediated combination chemotherapy and PDT using doxorubicin and methylene blue had a therapeutic potential (Khdair et al., 2010). Canti *et al.* investigated the effects of PDT with photoactivated aluminum disulfonated phthalocyanine combined with Adriamycin (doxorubicin) and cisplatinum on murine tumors. Mice with leukemia and lymphoma were treated with Adriamycin or cisplatinum and then with PDT. Combination of antiblastic drugs with PDT showed an additive anti-tumor effect. Therefore, such a combination was suggested to have the potential to decrease the effective doses of antiblastic drugs, as well as reducing toxic effects on normal tissues (Canti et al., 1998). Kästle *et al.* analyzed the effects of combination of PDT with heme oxygenase I and poly (ADP-ribose) polymerase inhibitors on melanoma cells in comparison to nonmalignant keratinocytes (Kastle et al., 2011). The researchers concluded that heme oxygenase I and poly (ADP-ribose) polymerase inhibitors could augment the efficiency of PDT (Kastle et al., 2011).

In addition, combination of PDT with anti-angiogenic drugs may also prove to be useful (Bhuvaneswari et al., 2009). For this purpose, anti-angiogenic monoclonal antibodies may be utilized. Hypoxia is an important feature of several solid tumors and can cause induction of angiogenesis (Gilkes et al., 2014). Zhou *et al.* reported that efficiency of PDT could be increased by anti-angiogenic therapy (Zhou et al., 2005). Ferrario *et al.* demonstrated that combination of anti-angiogenic treatment and PDT enhanced tumoricidal effects in comparison to individual treatments in mice (Ferrario et al., 2000). In another study, Ferrario *et al.* showed that bevacizumab (a monoclonal antibody against vascular endothelial growth factor A [VEGF-A]) could augment the efficiency of PDT (Ferrario and Gomer, 2006; Los et al., 2007). They suggested that inhibitors of VEGF have a potential to improve the clinical efficacy of PDT (Ferrario and Gomer, 2006). Similarly, Bhuvaneswari *et al.* reported that combination of hypericin mediated PDT and bevacizumab improved tumor response (Bhuvaneswari et al., 2007). In addition, another study showed that combination of PDT with anti-angiogenic treatment resulted in inhibition of tumor growth and improved survival in mice in comparison to individual treatments (Jiang et al., 2008). Thus, such a combination approach can be promising in terms of improving clinical results in glioblastoma (Jiang et al., 2008).

### PDT Combined With Radiotherapy

Allman *et al.* reported that combination of 5-aminolaevulinic acid mediated PDT and gamma-irradiation caused a level of cytotoxicity which is additive and not synergistic (Allman et al., 2000). Another study by Luksiene *et al.* showed that hematoporphyrin dimethyl ether mediated PDT and radiotherapy resulted in the inhibition of tumor growth. Furthermore, combining PDT and radiotherapy yielded an additive effect (Luksiene et al., 1999). Nakano *et al.* reported that the treatment rate of Bowen’s disease might be enhanced via combination therapy with 5-aminolevulinic acid mediated PDT and radiation therapy (Nakano et al., 2011). The combinations of two of the modalities of Photofrin II-sensitized photochemotherapy, an electric current and ionizing radiation were reported to act mainly additively or synergistically (Ma et al., 1993).

### Photodynamic Therapy Combined With Surgery

Combinations of PDT with surgery have been studied by several groups. Rigual *et al.* proposed that adjuvant use of 2-(1-hexyloxyethyl)-2-devinyl pyropheophorbide-a (HPPH) mediated PDT and surgery for head and neck squamous cell carcinoma appeared safe (Rigual et al., 2013). Caesar *et al.* suggested that *m*-tetrahydroxyphenylchlorin (mTHPC) mediated PDT may be utilized as adjuvant therapy to surgery in recurrent tumors of the paranasal sinuses and the anterior skull base where complete resection is not achievable (Caesar et al., 2015). In another study, Doeveren *et al.* used adjuvant *meta*-tetrahydroxyphenylchlorin-mediated PDT in patients with a malignancy in the head and neck with close or positive resection margins who were not eligible for conventional treatment modalities and they reported that PDT could be applied as adjuvant therapy after surgery (van Doeveren et al., 2018). Wang *et al.* recently proposed that in prostate cancer, PDT might be an effective adjuvant therapy for image-guided surgery (Wang X. et al., 2020). Friedberg *et al.* reported that Foscan mediated PDT may be safely combined with surgery at the established maximally tolerated dose (Friedberg et al., 2003). Friedberg *et al.* conducted a phase II trial in order to investigate the effects of combination of surgery with intraoperative PDT on local control and survival in patients with non-small-cell lung cancer with pleural spread. They reported that surgery and PDT might be performed safely with good local control (Friedberg et al., 2004). Sun *et al.* suggested that PDT with photofrin on young patients with advanced colorectal cancer could be utilized as an adjuvant therapy (Sun et al., 2016). Kuijpers *et al.* reported four cases of basal cell carcinoma treated with Mohs surgery, where they used PDT with aminolevulinic acid as an adjuvant therapy (Kuijpers et al., 2004). The researchers proposed that the combination of Mohs surgery and PDT might result in the cure of the tumor with good cosmetic results (Kuijpers et al., 2004). Torres *et al.* conducted a preliminary study which found that methyl-aminolevulinate mediated PDT can be an alternative as an adjunctive therapy before standard surgical excision of morpheaform basal cell carcinoma (Torres et al., 2011). In another study, Nanashima suggested that adjuvant PDT may be a useful option for patients with bile duct carcinoma undergoing surgical resection (Nanashima et al., 2004). Hans-Beat Ris suggested that intraoperative PDT following resection can be an attractive treatment for malignant pleural mesothelioma, which may help to reduce local recurrence (Ris, 2005).

### Photodynamic Therapy Combined With Immunotherapy

PDT may also be utilized in combination with immunotherapy. It has the potential to augment anti-tumor immunity (Canti et al., 2010). It is known that generation of effective systemic anti-tumor immune responses has a great potential to eliminate tumors (Bonavida and Chouaib, 2017; Gonzalez et al., 2018; Labani-Motlagh et al., 2020; Hiam-Galvez et al., 2021). PDT is capable of inducing immunogenic cell death, which is a cell death modality that stimulates immune responses against dead cell antigens (Green et al., 2009; Kroemer et al., 2013; Ng et al., 2018). Furthermore, combining PDT with cancer immunotherapy may demonstrate synergistic results, achieve tumor regression, provide immune memory (Ng et al., 2018). In addition to such mechanisms related with immunogenic cell death achieved by PDT, treatment of tumors with infrared laser as a means of autologous vaccination has demonstrated promise in animal studies (Chen et al., 1999; Chen et al., 2001; Chen et al., 2003; Naylor et al., 2006). Thus, the release of tumor antigens can then play roles as *in situ* auto-vaccines (Naylor et al., 2006; Chen et al., 2016). Furthermore, proinflammatory cytokines, which stimulate the immune responses, are also increased. As a result of such mechanisms, PDT causes an increased dissemination of tumor antigens and damage associated patterns from the target tumors. The antigens are then taken up by dendritic cells, which subsequently present those antigens to CD4^+^ and CD8^+^ T cells. This presentation process results in the activation of adaptive immune responses against the tumor as well as generating immunological memory (Brackett and Gollnick, 2011; Reginato et al., 2014; Maeding et al., 2016; Murata et al., 2016; Alzeibak et al., 2021).

PDT is known to cause inflammation and recruitment of cells such as neutrophils, macrophages (Nowis et al., 2005). Jalili *et al.* investigated the effectiveness of combining PDT with administration of dendritic cells (Jalili et al., 2004). They reported that administration of immature dendritic cells into the tumors treated with PDT caused effective homing to lymph nodes and stimulated T and natural killer cells (Jalili et al., 2004). Thus, the researchers suggested that combination of PDT and administration of immature dendritic cells might demonstrate a potential for clinical use (Jalili et al., 2004). In another study, Saji *et al.* analyzed whether PDT followed by intratumoral administration of naive dendritic cells might stimulate anti-tumor immunity. They reported that PDT and intratumoral injection of dendritic cells resulted in systemic anti-tumor immunity in mice (Saji et al., 2006). PDT and low dose cyclophosphamide were shown to generate anti-tumor immunity in a study, which suggested that low dose cyclophosphamide depleted regulatory T cells and potentiated PDT (Castano et al., 2008). It was demonstrated that the combination of vinorelbine, PDT with *meta*-tetrahydroxyphenylchlorin and immune lymphocytes had a significant synergistic anti-tumor effect (Canti et al., 2010). In addition, similar results were obtained with the combination of cisplatin, PDT and immune lymphocytes (Canti et al., 2010).

Another interesting approach to combine PDT and immunotherapy relies on the generation of cancer vaccines by PDT, since such an approach has a potential to present tumor antigens (Korbelik, 2010). The PDT vaccine can be generated *ex vivo*/*in situ*. The vaccine can then be administered in order to eliminate tumor cells (Korbelik, 2019). Korbelik and Sun generated a whole tumor cell vaccine by *in vitro* PDT with benzoporphyrin derivative. They proposed that PDT may be utilized in order to generate efficient tumor vaccines (Korbelik and Sun, 2006). Gollnick *et al.* investigated PDT-generated murine tumor cell lysates and found that PDT-generated tumor cell lysates could be effective vaccines. In addition, they also reported that such vaccines were more efficient than those generated by UV or ionizing irradiation (Gollnick et al., 2002). Garg *et al.* combined dendritic cell immunotherapy and immunogenic cell death induced by PDT with hypericin. They suggested that immunogenic cell death based vaccines have the potential for translation to the clinical setting (Garg et al., 2016). In another study, Zhang *et al.* reported that 5-aminolevulinic acid mediated PDT dendritic cell vaccine might stimulate immune responses against tumors (Zhang H. et al., 2018). Similarly, Trempolec *et al.* reported that a PDT dendritic cell vaccine resulted in a considerable increase in IFNγ^+^ T cells and suggested that PDT dendritic cell vaccines may stimulate anti-tumor immune responses (Trempolec et al., 2020). Korbelik *et al.* suggested that surgically removed tumor tissues may be utilized for PDT-based vaccines. Such an approach would allow for the customization of therapeutic interventions (Korbelik et al., 2007). Thus, PDT-based vaccines hold the potential for precision medicine applications. Doix *et al.* investigated the effects of the dose of PS and PDT scheduling on immunity. Their findings suggested that the timing for the administration of PDT dendritic cell vaccine was important, since the administration of the vaccine before radiotherapy failed to increase tumor growth inhibition, whereas administration of the vaccine around/near radiotherapy resulted in considerable delay in tumor growth (Doix et al., 2019). A recent study reported that the semisynthetic biopolymer N-dihydrogalactochitosan increased the effectiveness of PDT tumor vaccines (Korbelik et al., 2019). Future studies about tumor vaccine related mechanisms are required, in order to be able to translate such approaches to the clinical use.

**FIGURE 7:**
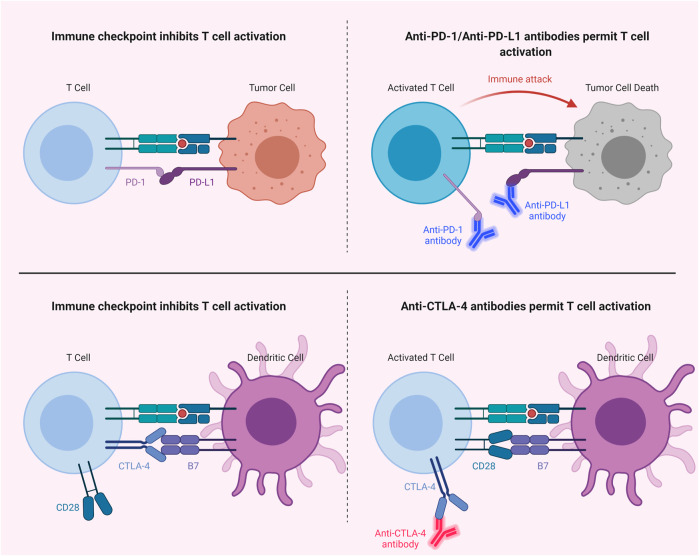
Immune Checkpoint Inhibition. Blocking of PD-1/PD-L1 or CTLA-4 with antibodies permits T cell activation. CTLA-4, cytotoxic T lymphocyte-associated protein 4; PD-1, programmed cell death protein 1; PD-L1, programmed cell death 1 ligand 1.

The effects of PDT on immune responses may in fact synergize with the effects of immunotherapeutic applications, such as the approaches aimed at attenuating the immune suppression in the tumor *milieu* via utilizing immune checkpoint inhibitors (*e.g.*, anti-PD-L1, anti-PD-1 and anti-CTLA-4) (Kleinovink et al., 2017; Cramer et al., 2020; Xu et al., 2020). Mechanisms of immune checkpoint inhibition can be seen in Figure 7. Immune checkpoint inhibition has the potential to induce tumor infiltrating lymphocytes (Darvin et al., 2018; Robert, 2020; Park et al., 2021). Given the fact that anti-tumor immune responses may be suppressed due to the effects of tumor cells on T cells via the programmed cell death 1 ligand 1 (PD-L1)/programmed cell death protein 1 (PD-1) immune checkpoint axis, PDT mediated cancer immunotherapy may be enhanced through targeting PD-L1 in tumor cells (Han et al., 2020; Lotfinejad et al., 2020). Wang *et al.* reported that combining PDT with PD-L1 knockdown demonstrated higher efficiency in terms of suppressing tumor growth and distant metastasis compared to only-PDT in a murine melanoma xenograft model (Wang et al., 2016). In another study, Dai *et al.* showed that PDT combined with PD-L1-blockade siRNA approach can stimulate a PDT-induced immune response and may be effective against immune resistance mediated by PD-L1 (Dai et al., 2018). He *et al.* demonstrated that PDT with nanoscale coordination polymer core-shell nanoparticles that carry oxaliplatin in the core and the PS pyropheophorbide-lipid conjugate in the shell combined with anti-PD-L1 suppressed the growth of both primary and distant tumors in murine colorectal cancer models (He et al., 2016). Duan *et al.* showed that PDT with Zn-pyrophosphate nanoparticles loaded with the PS pyrolipid sensitized tumors to checkpoint inhibition by a PD-L1 antibody. The approach could eliminate primary breast tumors and prevent lung metastasis (Duan et al., 2016). In another study, PDT with Fe-TBP was reported to enhance the efficiency of anti-PD-L1 treatment in a mouse colorectal cancer model (Lan et al., 2018). In addition, it induced abscopal effects. Moreover, PDT with Fe-TBP induced tumor cytotoxic T cell infiltration (Lan et al., 2018). Moreover, Santos *et al.* reported a favorable outcome in a clinical case with head and neck cancer, utilizing PDT with Redaporfin followed by an anti-PD-1 antibody (Santos et al., 2018).

In addition to the PD-1/PD-L1 axis, blockade of cytotoxic T lymphocyte-associated protein 4 (CTLA-4) checkpoint has also been studied in combination with PDT (Meng et al., 2019; Liu et al., 2021). Xu *et al.* reported that an immune-stimulating upconversion nanoparticle-based PDT approach in combination with CTLA-4 checkpoint blockade could effectively target primary tumors and suppress distant tumors, as well as inducing immunological memory (Xu et al., 2017). Furthermore, combination of PDT with indoleamine-2,3-dioxygenase (IDO) targeting may also prove to be a useful approach to stimulate immunity (Song et al., 2018); since, IDO is an enzyme which shows an immunosuppressive effect via tryptophan depletion, thereby helping tumors to escape from immunity (Moon et al., 2015). In addition, Lu *et al.* observed higher T cell infiltration in the tumor microenvironment with the combination of IDO inhibition and PDT induced immunogenic cell death (Lu et al., 2016). In light of the current literature, the combination of PDT and immunotherapy may not only efficiently eliminate the target tumor; but also can take out tumor cells and metastases. Such an approach can also provide immunological memory to preclude recurrence.

Even though a myriad of studies reported favorable outcomes in *in vitro* and *in vivo* investigations, further studies are required for the effective combination of PDT with other therapeutic modalities in the clinic.

## Photodynamic Therapy in Different Types of Cancers

As a noninvasive treatment, PDT is mostly utilized for the treatment of nonmelanoma skin cancer as well as precancerous lesions. In addition, PDT may also be utilized in order to treat internal tumors in several parts of the body. Such applications are achieved via administering a systemic PS and then applying specific light via an optical fiber (MacCormack, 2006). Indeed, various clinical applications in the fields of dermatology, gastroenterology, neurosurgery, pneumology, gynecology, urology and ophthalmology may demonstrate promising outcomes for the treatment of tumors (Verger et al., 2021).

### Photodynamic Therapy in Colorectal *Cancer*

Colorectal cancer is the third most common type of cancer with high invasive character and metastatic potential. Therefore, it constitutes an important clinical issue in which alternative treatments are investigated in addition to conventional therapies. There exist several PDT trials based on ALA and photofrin in colorectal cancer. In addition to these PDT approaches, novel treatment strategies are also being investigated (Kawczyk-Krupka et al., 2015).

The opinion that cancer stem cells are formed due to various genetic or epigenetic changes in adult normal stem cells in the human body has become an important perspective in recent years (Chaffer and Weinberg, 2011; Kreso and Dick, 2014; Ajani et al., 2015). In order to achieve this transformation, various genetic mutations and epigenetic modifications should be acquired. Although the cancer stem cell populations differ according to cancer type, they are very scarce (on average 0–2%) in the cancer tissue compared to other cell populations. Increased cancer stem cell proliferation in cancerous tissue shows a positive correlation with cancer’s aggressive character (Dalerba et al., 2007; Quintana et al., 2008; Eppert et al., 2011; Islam et al., 2015). It is stated that targeting cancer stem cells with PDT-mediated active or passive targeting strategies in colorectal cancer may be an important treatment approach, especially in metastatic colon cancer (Hodgkinson et al., 2017). In a study conducted on a colon cancer mouse model, it is stated that the cells undergo apoptosis as a result of PDT with chlorin-based nanoscale metal-organic framework (Lu et al., 2015). In addition, the combined use of PDT with chemo-radiotherapy may improve overall survival in advanced stage of cholangiocarcinoma (Triesscheijn et al., 2006; Yanovsky et al., 2019).

### Photodynamic Therapy in Lung *Cancer*

Studies on PDT in lung cancer have been carried out for several years. PDT alone was reported to be an important alternative treatment to palliative chemotherapy or radiotherapy in lung cancer. It results in a response rate of approximately 87% as well as improving the patient’s quality of life (Wang et al., 2021). In a study conducted on A549 lung cancer cells, PDT mediated with a nanobody complex (Nb@IC-NPs) was able to effectively destruct tumor cells and improve survival in mice (Zhang et al., 2020). PDT can be used in combination with chemotherapy, radiotherapy and surgery to treat non-small-cell lung cancer. It is proposed that PSs such as temoporfin, 2-[1-hexyloxyethyl]-2-devinyl pyropheophorbide-a, and chlorin e6 demonstrate more favorable toxicity profiles (Triesscheijn et al., 2006; Yanovsky et al., 2019). It was suggested that Photofrin-PDT might be useful as a palliative treatment in lung cancer. However, the efficacy of this method decreases in advanced cancers. It is proposed that current PSs such as talaporfin and HPPH, which have high absorption at longer wavelengths, are more effective than the first-generation photofrin (Allison et al., 2011; van Straten et al., 2017; Yang et al., 2021).

### Photodynamic Therapy in Prostate *Cancer*

Prostate cancer is the most frequent cancer in males and it is the second leading cause of cancer death in men in United States (Siegel et al., 2020). Conventional therapies may cause prominent side effects. For this reason, focal therapies such as cryotherapy or PDT are important (Gheewala et al., 2017). Although padoporfin and motexafin lutetium (MLu) based PDTs may show effective results, it has been noted that PDT approaches used in prostate cancer treatment have various toxic effects. Therefore, it is necessary to revise the treatment strategy by performing dosimetric analyses (Agostinis et al., 2011; Moore et al., 2011).

### Photodynamic Therapy in Head & Neck *Cancer*

Head and neck cancer is responsible from more than 650,000 cases and 330,000 deaths annually worldwide (Bray et al., 2018; Stenson, 2021). Temoporfin-based PDT can be used as a treatment method in advanced-stage head and neck cancers when surgery and radiotherapy may be insufficient (Li X. et al., 2020). Photofrin PDT, which is used in patients diagnosed with nasopharyngeal carcinoma and relapsed due to conventional treatments such as fluorouracil and cisplatin, has been reported to be more effective than conventional treatment (Li et al., 2006; Agostinis et al., 2011).

### Photodynamic Therapy in Brain *Cancer*

Malignant gliomas may be fatal tumors. Additionally, post-treatment recurrence rates are approximately 80%. Current treatment strategies result in an overall survival of about 15 months (Quirk et al., 2015). PSs can be used for both PDD and PDT purposes in brain tumors. It was reported that ALA-based malignant glioma surgery could increase overall survival compared to the control group (Stummer et al., 2006; Agostinis et al., 2011).

### Photodynamic Therapy in Skin *Cancer*

PDT is frequently used in skin diseases, especially in nonmelanoma skin cancer and precancerous cutaneous lesions. Over 10 million patients have been reported to be treated with PDT so far (Li X. et al., 2020). PDT is generally preferred in the treatment of actinic keratosis (AK). It was stated that ALA-based PDT is a more effective treatment strategy compared to the cryotherapy used in AK treatment. It was reported that MAL-PDT (methyl aminolevulinate) applied in the treatment of squamous cell carcinoma *in situ* might achieve 88–100% elimination of the lesions. Although the efficiency of PDT treatment in basal cell carcinoma is similar to cryotherapy and surgical application, PDT provides easy application for difficult areas such as eye lesions (Triesscheijn et al., 2006; Yanovsky et al., 2019).

### Photodynamic Therapy in Pancreas *Cancer*

Pancreas cancer is another type of cancer with high fatality. The 5-years survival in pancreas cancer is less than 10%. Current therapeutic strategies may cause adverse effects (Wang Y. et al., 2020). For this reason, various clinical and preclinical trials are ongoing in the setting of pancreas cancer. Phase studies with benzoporphyrin-based PDT in pancreas cancer may yield results associated with low morbidity (van Straten et al., 2017; Yang et al., 2021). Bown *et al.* utilized PDT with meso-tetrahydroxyphenyl chlorin in pancreas cancer (Bown et al., 2002). They reported that PDT could produce necrosis in pancreatic cancers with an acceptable morbidity. In another study, Huggett *et al.* suggested that Verteporfin PDT-induced tumor necrosis is feasible and safe in locally advanced pancreatic cancer (Huggett et al., 2014).

### Photodynamic Therapy in Breast *Cancer*

Breast cancer is the most common cancer among women. It demonstrates a complex genetic basis in terms of susceptibility. As a result of the treatment strategies applied for breast cancer, adverse effects and resistance to treatment may occur in some breast cancer subtypes. The new treatment strategies include biomimetic hydrogels encapsulating ROS - sensitive tegafur (TF) and protoporphyrin IX (PpIX) heterodimers (TTP), which aim to utilize chemotherapy and PDT synergistically in order to minimize the side effects that occur during conventional treatment (Aniogo et al., 2019; Zhang et al., 2021).

### Photodynamic Therapy in Cervix *Cancer*

Cervical cancer is the fourth most common cancer in women (Bray et al., 2018; Arbyn et al., 2020). It is estimated that cervical cancer accounted for 604,000 new cancer cases and 342,000 deaths worldwide in 2020 (Sung et al., 2021). Although conventional treatments have effective responses, cervical stroma is damaged in most patients due to these treatments. In this context, PDT emerges as an effective and harmless treatment strategy (Li D. et al., 2020). Phase studies with ALA-based PDT in cervical cancer have yielded results associated with low morbidity (Allison et al., 2011; van Straten et al., 2017; Yang et al., 2021). A study conducted with HeLa cells showed that cellular uptake increased with phthalocyanine conjugated gold nanoparticles. In addition, the caspase 3/7 pathway was suggested to be activated in the cells as a result of PDT (Wieder et al., 2006).

## Concluding Remarks

Various novel PDT approaches have been studied and developed over the last decades in order to be used against tumors. Preclinical and clinical applications of PDT and PDD have yielded promising results. Topical and systemic administrations of PSs have been utilized in the clinical setting. In addition, several PDT approaches have been approved by major regulatory bodies including the FDA in the United States and the European Medicines Agency (EMA) in Europe. Although we have witnessed a considerable progress in understanding the mechanisms associated with PDT and PDD, the systemic applications of PSs still require further developments. Furthermore, the biophysical features of the constituents of PDT (*i.e.*, PS, light, oxygen) may still represent limitations for the clinical applications of PDT, especially for deep tissue seated hypoxic tumors. Targeted approaches especially in the setting of precision medicine may pave the way for more efficient and widespread utilization of PDT in the clinic. Numerous studies have concentrated on improving the effectiveness and specificity of PDT for specific tumor cells. Such studies demonstrated that various approaches including nanoparticles could be used to achieve such goals. In addition, tumor related cues may also be used as biomarkers. Thus, approaches that target such cues have the potential to improve the specificity of the treatment, as well as decreasing systemic adverse effects. Furthermore, the combination of PDT with other therapeutic modalities such as chemotherapy, immunotherapy, etc. has demonstrated favorable results. Various studies are ongoing in terms of the efforts to determine the optimal combination approaches. Moreover, preclinical interest in designing state-of-the-art PSs for use in PDT or PDD will facilitate the development of more capable and advanced agents. It should also be noted that there are several clinical trials in progress, currently. The importance of the ease of applicability and effectiveness of new treatment modalities in comparison to standard approaches should also be kept in mind. Reasonable strategies may be helpful for clinical translation of novel PDT modalities; thus, allowing for more common use of PDT. In addition to the development of more refined PSs, a better understanding of the effectiveness of PDT in a combination setting in the clinic as well as the optimization of such complex multimodal treatments will expand the clinical applications of PDT.

## Glossary

ADP: adenosine diphosphate
AK: actinic keratosis
ALA: aminolevulinic acid
ATP: adenosine triphosphate
CTLA-4: cytotoxic T lymphocyte-associated protein 4
ECM: extracellular matrix
EET: excitation energy transfer
EMA: european medicines agency
FDA: food and drug administration
GLUT: glucose transporter
HER2: human epidermal growth factor receptor 2
HpD: hematoporphyrin derivative
HPPH: 2-(1-hexyloxyethyl)-2-devinyl pyropheophorbide-a
IC: internal conversion
IDO: indoleamine-2,3-dioxygenase
IFNγ: interferon gamma
ISC: intersystem crossing
LED: light emitting diodes
MAL: methyl aminolevulinate
MDR: multidrug resistance
MLu: motexafin lutetium
MRI: magnetic resonance imaging
mTHPC: *m*-tetrahydroxyphenylchlorin
NIR: near-infrared
NSCLC: non-small-cell lung cancer
PD-1: programmed cell death protein 1
PDD: photodynamic diagnosis
PDK: pyruvate dehydrogenase kinase
PD-L1: programmed cell death 1 ligand 1
PDT: photodynamic therapy
PIT: photoimmunotherapy
PpIX: protoporphyrin IX
PS: photosensitizer
ROS: reactive oxygen species
UV: ultraviolet
VEGF: vascular endothelial growth factor
